# Measurement of Regional 2D Gas Transport Efficiency in Rabbit Lung Using Hyperpolarized 129Xe MRI

**DOI:** 10.1038/s41598-019-38942-8

**Published:** 2019-02-20

**Authors:** Kai Ruppert, Yi Xin, Hooman Hamedani, Faraz Amzajerdian, Luis Loza, Tahmina Achekzai, Ian F. Duncan, Harrilla Profka, Sarmad Siddiqui, Mehrdad Pourfathi, Federico Sertic, Maurizio F. Cereda, Stephen Kadlecek, Rahim R. Rizi

**Affiliations:** 1 Department of Radiology, University of Pennsylvania, Philadelphia, PA 19104 USA; 2 Department of Bioengineering, University of Pennsylvania, Philadelphia, PA 19104 USA; 3 Department of Electrical and Systems Engineering, University of Pennsylvania, Philadelphia, PA 19104 USA; 4 Department of Anesthesiology and Critical Care, University of Pennsylvania, Philadelphia, PA 19104 USA

## Abstract

While hyperpolarized xenon-129 (HXe) MRI offers a wide array of tools for assessing functional aspects of the lung, existing techniques provide only limited quantitative information about the impact of an observed pathology on overall lung function. By selectively destroying the alveolar HXe gas phase magnetization in a volume of interest and monitoring the subsequent decrease in the signal from xenon dissolved in the blood inside the left ventricle of the heart, it is possible to directly measure the contribution of that saturated lung volume to the gas transport capacity of the entire lung. In mechanically ventilated rabbits, we found that both xenon gas transport and transport efficiency exhibited a gravitation-induced anterior-to-posterior gradient that disappeared or reversed direction, respectively, when the animal was turned from supine to prone position. Further, posterior ventilation defects secondary to acute lung injury could be re-inflated by applying positive end expiratory pressure, although at the expense of decreased gas transport efficiency in the anterior volumes. These findings suggest that our technique might prove highly valuable for evaluating lung transplants and lung resections, and could improve our understanding of optimal mechanical ventilator settings in acute lung injury.

## Introduction

Hyperpolarized xenon-129 (HXe) MRI offers a wide array of techniques for assessing lung structure and function that can complement existing clinical modalities such as pulmonary function tests, blood gases, chest x-rays, and CT. For example, since pulmonary function tests and blood gases provide only global functional parameters, while x-ray and CT images yield mainly static structural information, existing HXe MRI techniques can measure the regional pulmonary gas exchange between the alveolar volume and the lung parenchyma, a parameter invisible to those conventional modalities. Other functional imaging methodologies have substantially evolved in the evaluation of regional lung function as well^1–3^. For instance, clinically employed alveolar ventilation/perfusion mapping can detect local mismatches between ventilation and perfusion, and positron emission tomography (PET) provides metrics of ventilation and perfusion that can also be used to calculate regional gas uptake^4^. However, no current technique is able to directly quantify the ability of a particular pulmonary region to transfer gas to the arterial blood and transport it to the body, a capability that HXe MRI has the potential to provide. Although differences between xenon’s physical and chemical properties and those of the respiratory gases oxygen and carbon dioxide lead to quantitatively different gas exchange kinetics, xenon does follow equivalent passive diffusion pathways from the alveolar airspaces to the hemoglobin inside the red blood cells. Thus, any pathological changes in pulmonary gas exchange should correlate well with similar changes in xenon gas exchange.

Xenon-129 has already shown promise as a tool for investigating pulmonary gas exchange processes—due to both its solubility in water and biological tissues^5^ and the fact that it exhibits a large change in resonance frequency of about 200 ppm downfield of the gas-phase (GP) resonance upon dissolution^6^. At an imaging field strength of 1.5 T, the chemical shift difference between GP xenon inside the pulmonary airspaces and dissolved-phase (DP) xenon in the parenchyma, blood and heart translates into a frequency difference of approximately 3.5 kHz, facilitating independent manipulation and observation of these two distinct spectral regions. So far, the gas uptake of xenon by the lung has been predominantly evaluated by imaging the DP distribution either in steady state^7–16^, dynamically as a function of time after DP saturation with a narrow bandwidth RF pulse^17–28^ or, more recently, as a combination of the two by analyzing multiple data sets acquired with different flip angles or flip angle – repetition time (TR) combinations^29,30^. Yet each region of the lungs contributes only an individual, unknown quota to the overall amount of gas transport from the alveoli to the arterial blood, which depends on the distributions of ventilation and blood flow, as well as the local efficacy of alveolar-capillary diffusion. Thus, while existing DP MRI techniques may be sufficiently sensitive to localize regional abnormalities in pulmonary gas exchange, they provide only limited information about the actual impact of an observed pathology on overall lung function.

The large chemical shift difference between GP and DP means that the dynamics of HXe accumulation in one or more tissue compartments of the lung can easily be quantified, but the measurement of actual net xenon transport by the pulmonary circulation is less straightforward. Upon inhalation, xenon quickly dissolves in the alveolar septal walls and capillary blood in proportion to their volume and the xenon solubility for each physiological compartment. Once these compartments are saturated with xenon, the xenon magnetization reaches steady state conditions. Despite continuous removal of xenon from the alveoli by the blood stream this volume is immediately replenished by fresh xenon from the alveolar GP such that the net DP magnetization appears to be approximately constant and the DP signal in any given pixel within the lung parenchyma will no longer reflect blood flow; however, even with non-equilibrium measurements, it is challenging to accurately extract directional transport processes. One way out of this dilemma is to quantify the xenon DP signal once it has left the lung parenchyma. Since essentially all xenon gas that is taken up by the blood stream passes through the heart before it is distributed throughout the body, the left atrium and ventricle represent excellent central reporting sites for this purpose. If the xenon DP in the heart following saturation of the xenon GP magnetization in a volume of interest is compared to the heart signal without GP saturation, the contribution of the saturated region to the total pulmonary gas transport can be determined in a completely non-invasive and model free manner^29^. In this work, we investigated the feasibility of such an approach by characterizing regional pulmonary gas transport in rabbits, both as a function of animal position and in a model of acute lung injury following acid aspiration. Although only a single DP resonance can be resolved in rabbits at 1.5 T, one advantage of our proposed method is that it is entirely imaging-based and does not depend on specifically identifying xenon bound to hemoglobin. It is therefore applicable in any species.

## Methods

### Animal Studies

Five New Zealand rabbits (3.5–4.5 kg) were anesthetized (intraperitoneal ketamine and xylazine) and tracheotomized. Peripheral veins were accessed to maintain general anesthesia (Propofol), and 15 ml/kg per hour of saline was given for hydration and to stabilize hemodynamics. Animals were mechanically ventilated using a custom-built ventilator, with FiO_2_ 0.3, tidal volume 6 ml/kg, and respiratory rate 40 breaths per minute, while body temperature was supported by a circulating warm water pad. Animals were euthanized at the end of the imaging procedures. All experiments were approved by and performed in accordance with the guidelines established by the University of Pennsylvania Institutional Animal Care and Use Committee and the NIH guidelines for the care and use of laboratory animals.

Images were obtained during breath holds at EE or EI respiratory phases. Animals were studied in five experimental settings: (1) To explore the impact of the GP saturation, one supine rabbit was scanned with the flip angle for saturating the GP in the right lung incremented from 15° to 120°; (2) To show the effect of subject orientation, one rabbit was studied in prone and supine position at PEEP 0 cm H_2_O; (3) To test the reproducibility of the measurements, a supine rabbit was imaged three times at EE with a PEEP of 0 cm H_2_O; identical sets of 4 GP saturation bands were created by incrementally shifting a 50 mm regional saturation slab from posterior to anterior in 1 cm steps; (4) To measure the impact of PEEP and respiratory phase, one rabbit was studied supine at both EI and EE with PEEP 0 cm H_2_O, and at EE with PEEP 5 and 10 cm H_2_O, with and without GP saturation; (5) To investigate the effects of mild focal injury, two rabbits received direct endo-bronchial instillation of hydrochloric acid (HCl, 0.75 ml/kg, PH 1.25) through a catheter (OD 1/16”) wedged into a bronchus. In this group, images were acquired before and after lung injury at PEEP 0 and 5 cm H_2_O.

### Gas Polarization and Administration

Enriched xenon gas (87% xenon-129) was polarized by collisional spin exchange with an optically pumped rubidium vapor using a prototype commercial system (XeBox-E10; Xemed, LLC, Durham, NH) that provided gas polarizations of 40–50%. Immediately before MR data acquisition, 1.25–1.5 L of HXe gas was dispensed into a Tedlar bag (Jensen Inert Products, Coral Springs, FL) inside a pressurizable cylinder that was subsequently connected to and controlled by the ventilator. At the beginning of the imaging study, animals were ventilated with 30% oxygen and 70% HXe (6 ml/kg tidal volume). After inhalation of the gas mixture for up to 3 breaths, ventilation was suspended for up to 7 s at either EI or EE for image acquisition.

### HXe Data Acquisition

Imaging was performed on a 1.5-Tesla commercial whole-body scanner (Magnetom Avanto; Siemens Medical Solutions, Malvern, PA, USA) that had been modified by the addition of a broadband amplifier to permit operation at the resonant frequency of 17.6 MHz. The RF coil was a custom xenon-129 transmit/receive birdcage design (Stark Contrast, Erlangen, Germany), positioned to cover the whole chest of the animal. Low-resolution proton MR scout images were obtained with the built-in body coil.

The RF excitation flip angle was calibrated during an initial 5 s breath hold, during which 32 xenon spectra were acquired. The TR for the first 16 acquisitions was set to 100 ms, and was extended to 200 ms for the second 16 acquisitions. Exponential decay functions where fitted to the integral of the GP amplitude in the phased, real spectra of both sets, and the T1-corrected flip angle was calculated^31^. The ratio between the nominal and the measured flip angle was used to set the reference voltage for all subsequent studies.

At 1.5 T, all xenon resonances in the rabbit lung are merged into a single peak separated from the GP resonance by approximately 200 ppm. To image these two frequency bands individually, we implemented a 2D projection acquisition similar to the one previously described in detail^8^. Briefly, the pulse sequence is based on a standard RF-spoiled gradient echo sequence with a non-selective 700-μs Gaussian RF excitation pulse centered 200 ppm downfield from the gas resonance that predominantly excites the DP region. However, the RF pulse was sufficiently short to excite the GP resonance as well, albeit with an amplitude 2.5% that of the DP resonance. This scaling relationship was established through a calibration acquisition that measured each k-space line twice: once with a 40° excitation pulse centered at the DP resonance, and once with a 2° excitation flip angle centered at the GP resonance. The GP signal for the 2° GP excitation was approximately twice as large as for the 40° DP excitation.

To destroy any DP magnetization taken up prior to the data collection and thereby achieve a steady state condition for the DP signal, the sequence was first preceded by two 2-ms Gaussian RF saturation pulses. Next, a series of 700-μs Gaussian RF excitation pulses was applied for 1.5 s using the same TR as the image acquisition. All preparatory RF pulses were also centered at 200 ppm. The sampling was 65% asymmetric with a bandwidth of 110 Hz, which, at the main field strength of 1.5 T and a gyromagnetic ratio for xenon-129 of 11.78 MHz/T, yielded a 32-pixel separation between the GP and DP images in the readout direction. Other sequence parameters included: matrix size 28 × 80 (interpolated to 112 × 320); TR/TE 200/2.6 ms; FOV 220–238 mm; flip angle at 200 ppm 30–40°. These flip angle – TR combinations offer a sufficiently low DP signal depolarization with each RF pulse and a sufficiently long delay time between consecutive RF pulses to ensure that enough DP magnetization can accumulate in the heart to provide a signal-to-noise ratio. The 30°/200 ms acquisition is equivalent to the application of a 90° RF pulse that would destroy the entire DP magnetization every TR_90°, equiv_ = 1.5 s (40°/200 ms; TR_90°, equiv_ = 0.9 s)^32^. Acquisitions with shorter TR but similar TR_90°, equiv_ are feasible albeit the price of a reduced measurement accuracy.

Each study consisted of an acquisition without GP saturation to generate a reference data set, followed by three identical acquisitions following a regional GP saturation at the beginning of the breath hold. Regional GP saturation was performed using a 50 mm saturation slab positioned along the anterior – posterior axis of the animal. The slab position was shifted twice in 10 mm increments between acquisitions. The flip angle of the GP RF saturation pulse was approximately 90°. To investigate the impact of RF saturation pulse flip angle on the DP signal change in the heart, the former was varied between 15° and 120°, in 15° increments, while the saturation slab covered the entire right lung.

### Data Analysis

All image reconstruction, post-processing and data analysis was performed using customized MatLab (Mathworks, Natick, MA, USA) scripts. The asymmetrically sampled k-space data was filled using a Homodyne algorithm^33^ before Fourier transform.

**Figure 1:**
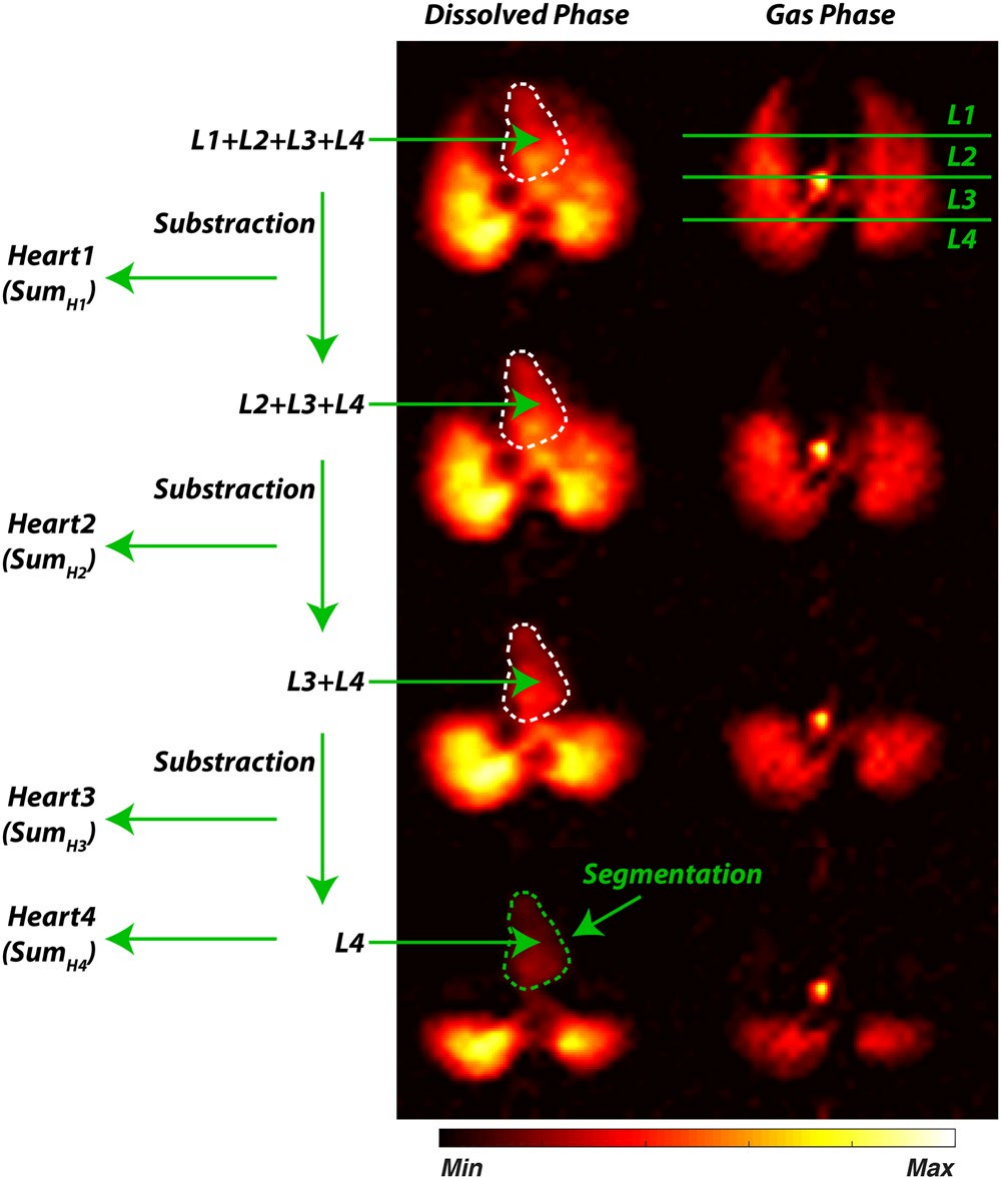
Schematic of the image analysis in a GP-saturation data set. The heart and aortic arch were manually segmented from the acquisition with the largest anterior GP saturation volume (bottom row), and the mask was applied to all other images in the set. The contributions of lung regions L1-L4 to the pulmonary gas transport were calculated as the difference in DP signal within the heart mask between consecutive acquisition pairs (top to bottom). The residual heart signal following the GP saturation with the largest volume was assigned to the remaining ventilated lung volume (bottom row).

A summary diagram of the image analysis is shown in Fig. 1. Within the DP image with the largest anterior GP saturation volume, the heart and the aortic arch were manually segmented. These segmentation masks were then applied to the other three DP images in the set, allowing a selective extraction of the xenon DP signal from these volumes. In one study, the manual segmentation was repeated by three different operators for each image in order to confirm the robustness of the procedure with respect to operator uncertainties. To obtain the ventilated volume, the left and right lungs were manually delineated in the non-saturated GP images, and the mask thus obtained was used to segment the lungs in the GP maps of the measurements acquired with regional GP saturation. The large airways were excluded from the segmentation.

All GP-saturated acquisitions were normalized to the unsaturated reference data by applying the GP segmentation mask of the GP-saturated acquisition to the reference measurement. The ratio of the median GP signals within these two masked images was then used to scale the GP-saturated images. To measure the contribution of each GP-saturated region to the total pulmonary gas transport, the difference ΔDP_H_ of the normalized DP signal inside the heart mask between the 4 acquisitions of a set was calculated. The functional efficiency of the regional gas transport in a lung volume was determined as the ratio between ΔDP_H_ and the corresponding difference of the total GP signal, respectively.

## Results

**Figure 2:**
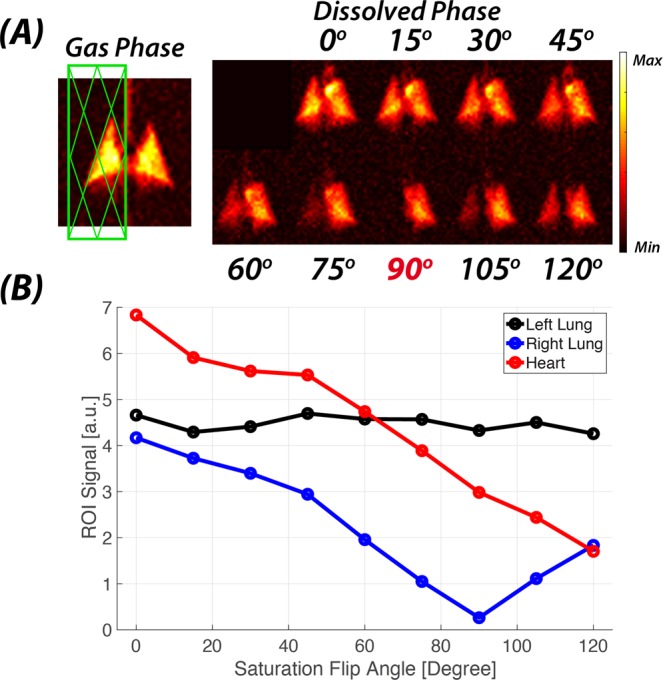
Impact of GP saturation pulse flip angle on DP magnetization in a slab covering the right lung. (**A**) Coronal DP projection maps following GP saturation with flip angles ranging from 0° (no saturation) to 120°. (**B**) Total DP signal in left lung, right lung and left heart regions based on maps in (**A**) as a function of GP saturation flip angle.

Figure 2 demonstrates the response of the DP magnetization to the application of an RF saturation pulse selective for the right lung (green box in left-hand panel of Fig. 2A). The residual GP signal within the saturation slab is proportional to the cosine of the flip angle of the saturation pulse such that, for instance, an ideal 90° flip angle would destroy the entire GP magnetization inside the slab while leaving the GP magnetization outside the slab untouched. Any subsequently acquired maps of the DP magnetization distribution will then reflect the pulmonary gas uptake and transport of the manipulated GP magnetization distribution. This effect is shown by increasing the flip angle of the GP saturation of the right lung from 0° to 120°, which changed the intensity of the 2D-projection DP signal in both the parenchyma of that same volume and in the downstream vasculature and left heart (Fig. 2A). No changes in the DP signal within the parenchyma of the left lung were observable. In Fig. 2B, the DP amplitudes for the left and right lungs as well as the heart are plotted as a function of the flip angle of the GP RF saturation pulse. As expected, the DP signal behavior of the right lung approximated a cosine curve with a minimum at 90°. However, the DP signal in the heart continued to decline up to the maximum achievable flip angle of 120°.

**Figure 3:**
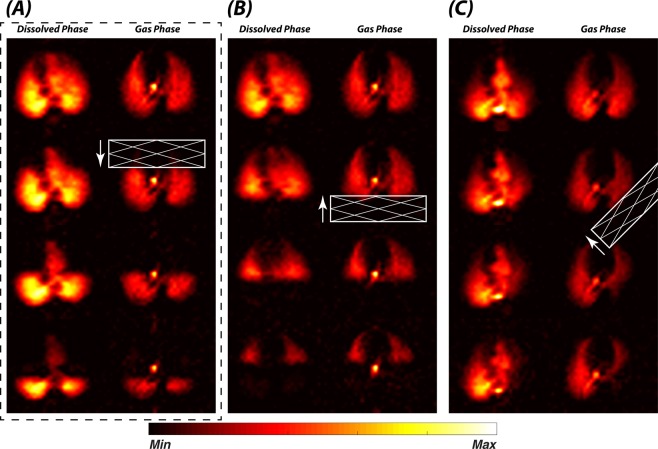
Axial DP-GP projection maps following GP saturation in incrementally shifted slabs. Examples of advancing saturation slabs along the (**A**) anterior-to-posterior, (**B**) posterior-to-anterior, and (**C**) left-posterior to right-anterior direction.

Figure 3 illustrates how pulmonary xenon gas transport can be assessed by incrementally shifting the position of the GP saturation slab across the lung: upon saturation of their GP magnetization, lung volumes that made large contributions to the pulmonary gas transport reduced the DP signal in the heart more significantly than volumes with small contributions. In supine rabbits, saturation of the posterior GP in particular diminished the heart signal so drastically that subsequent saturation increments were difficult to analyze. Regardless of animal orientation, the GP saturation steps were therefore conducted in ventral-to-dorsal direction in all subsequent studies.

**Figure 4:**
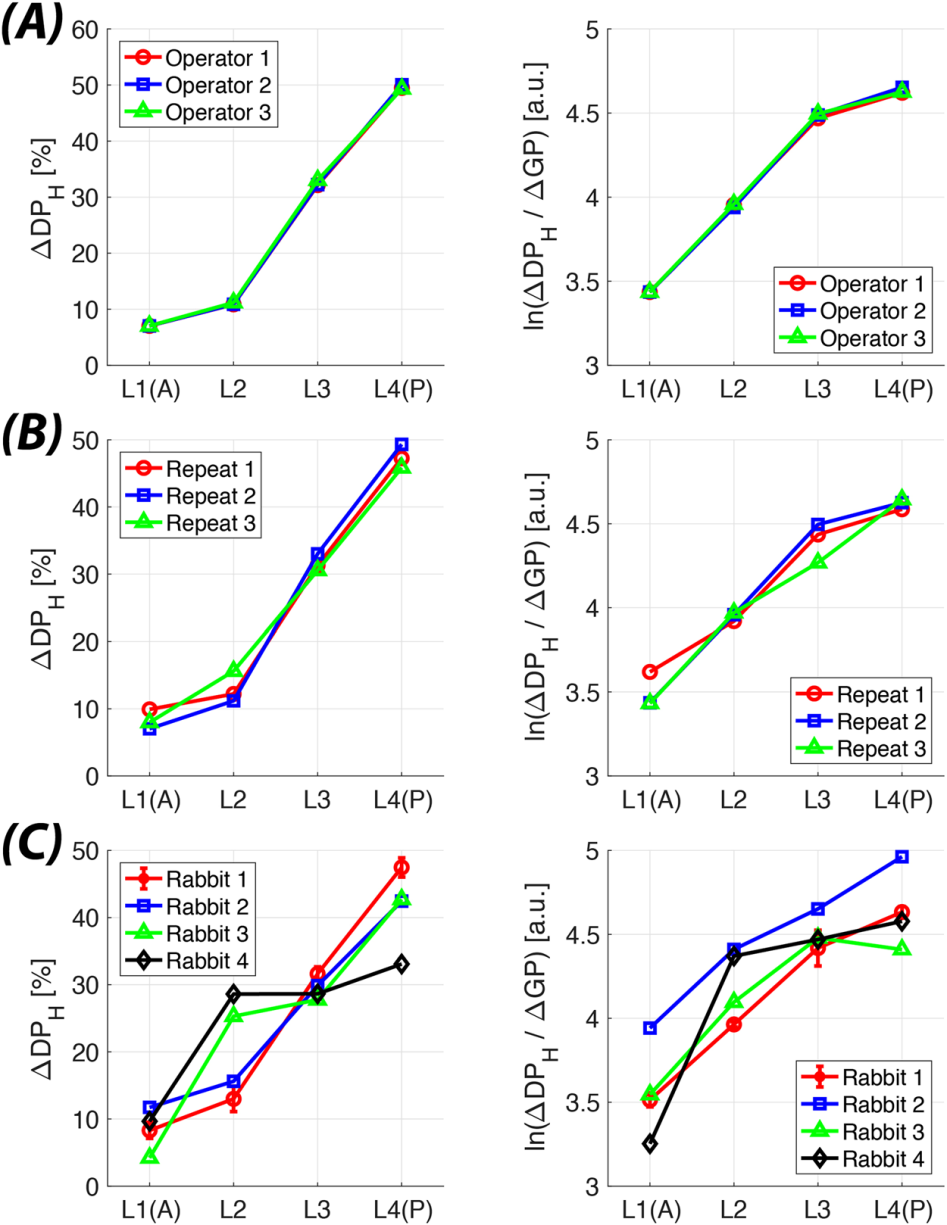
Intra-subject measurement variability at EE in supine rabbits. Regional gas transport (left column) and transport efficiency (right column) for (**A**) segmentation of the heart and lungs by three different operators, and (**B**) three consecutive repeat measurements in the same animal.

The repeatability of the segmentation process was tested preliminarily by manually segmenting the heart and lungs three times without finding any significant differences in segmentation size of the heart (64.7 ± 2.1 pixels) or the numerical results (Fig. 4A). Slightly larger variations were seen when the same sets of GP saturation bands were applied three times in the same animal (Fig. 4B). A more robust repeatability test will be conducted as our measurements are performed in additional animals under a variety of study conditions.

**Figure 5:**
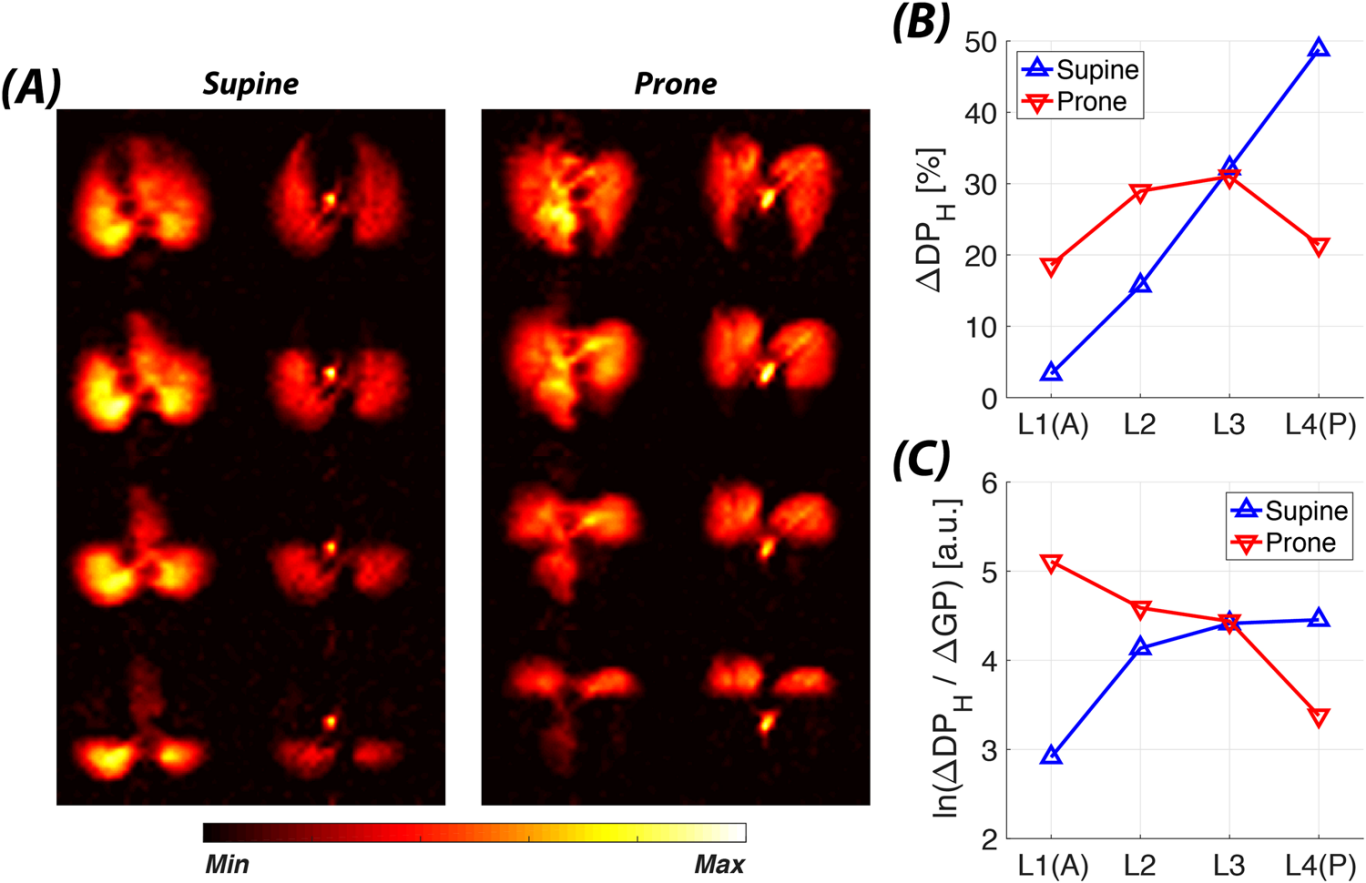
Orientation-dependent changes in the pulmonary gas transport. (**A**) Axial DP-GP projection maps for a rabbit in supine (left panel) or prone (right panel) position. (**B**) Regional contributions to the total xenon gas transport in supine (red line) or prone (blue line) position. (**C**) Semi-log plot of regional xenon gas transport efficiency in supine (red line) or prone (blue line) position.

To further evaluate the sensitivity of our technique through comparison to known physiological responses^34^, we investigated the gas transport behavior of a rabbit in supine versus prone orientation (Fig. 5). In supine position, a strong vertical gradient of ΔDP_H_ was observed, with the most dependent (dorsal) saturation volume contributing almost 50% of the total transport—approximately five times as much as the most non-dependent volume. This gradient largely disappeared when the animal was turned prone (Fig. 5B), and all four saturation volumes contributed between 20% and 30%. However, the direction of the gas transport efficiency gradient was reversed between supine and prone positioning, with ventral predominance in the prone position (Fig. 5C).

**Figure 6:**
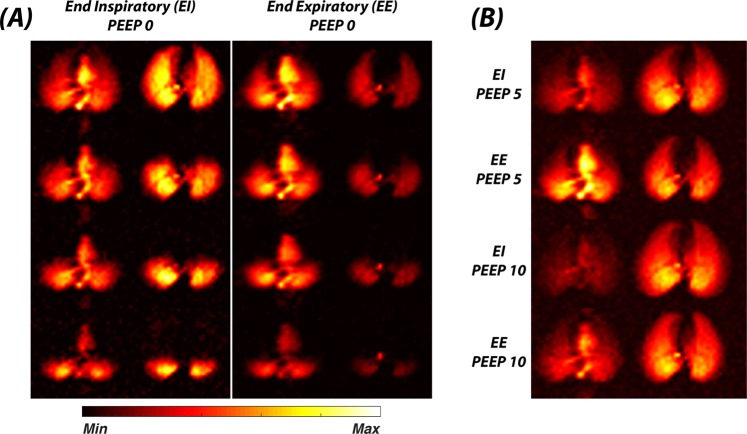
Impact of lung inflation pressure on xenon gas transport. (**A**) Axial DP-GP projection maps for a breath hold at PEEP 0 cm H_2_O and EI (left panel) or EE (right panel). (**B**) Axial DP-GP projection maps for a breath hold at (from top to bottom) PEEP 5 cm H_2_O and EI, PEEP 5 cm H_2_O and EE, PEEP 10 cm H_2_O and EI, PEEP 10 cm H_2_O and EE. Higher intrapulmonary pressure lowers the DP signal.

**Figure 7:**
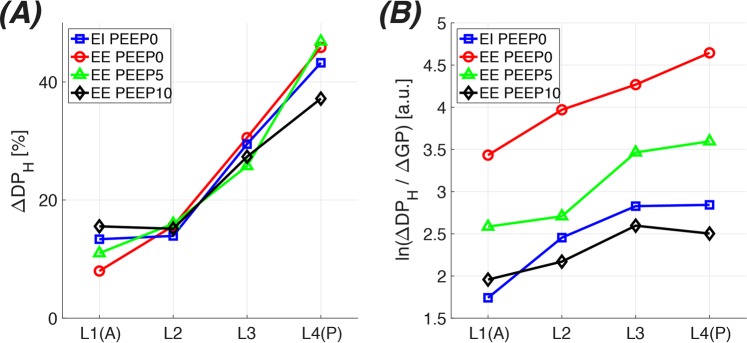
Regional contributions to the total xenon gas transport (**A**) and semi-log plots of transport efficiency (**B**) as a function of lung inflation during breath hold.

Figure 6 depicts the impact of respiratory phase (end-expiratory, EE, versus end-inspiratory, EI) and applied positive end-expiratory pressure (PEEP) on the distribution of the DP signal in the lungs of supine rabbits. Unsurprisingly, higher associated intrapulmonary pressure caused a larger cross section of the lung to appear in the GP and DP 2D projection images. In contrast, the apparent size of the left heart and the normalized DP signal intensities showed an inverse relationship to intrapulmonary pressure. In particular, EI measurements with PEEP resulted in such low DP signals in the heart that the impact of regional GP saturation could not be reliably quantified. These measurements were therefore excluded from further analysis. The quantitative results for the remaining experiments in Fig. 6 are displayed in Fig. 7. Although no explicit pressure readings were available, the EI PEEP 0 cm H_2_O measurement slotted in-between the EE PEEP 5 cm H_2_O and EE PEEP 10 cm H_2_O experiments based on both gas transport contribution and efficiency curves. The absolute gas transport efficiency decreased by a factor of approximately 7 throughout the lung as intrapulmonary pressure rose. At the regional level, the contribution to gas transport and efficiency of the anterior-most saturation segment increased, but both parameters declined in the posterior-most segment. However, interpretation of this latter aspect was confounded by the fact that the absolute location of the GP saturation slabs was fixed while the lung expanded with pressure.

**Figure 8:**
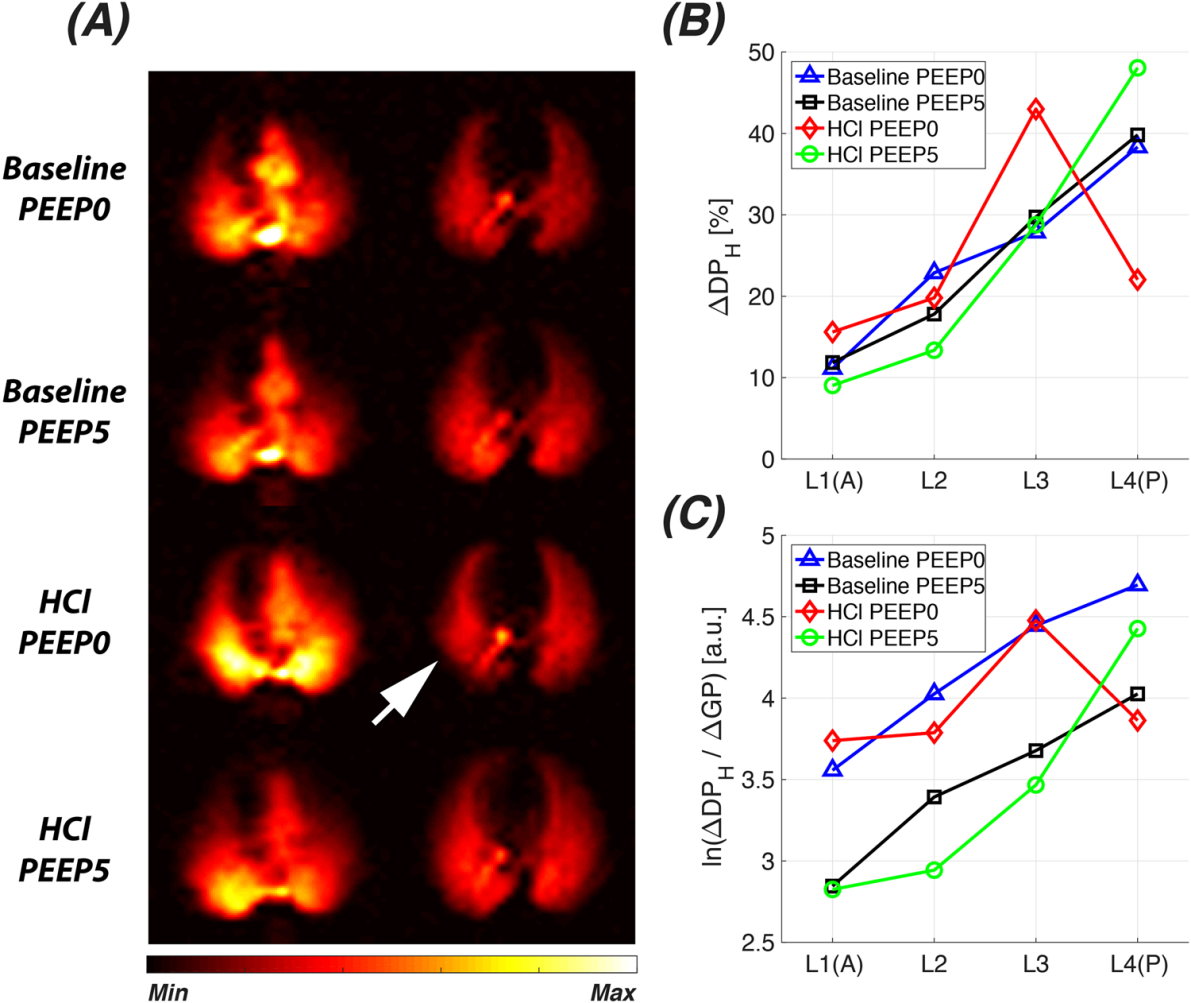
Impact of acute lung injury on pulmonary gas transport in a rabbit model of pulmonary acid aspiration with and without PEEP. (**A**) Axial DP-GP projection maps for a rabbit at baseline (top 2 rows) and approximately 1 hour after HCl instillation (bottom 2 rows). (**B**) Changes in the regional contributions to the total xenon gas transport for the measurements in (**A**). (**C**) Changes in semi-log plots of regional xenon gas transport efficiency for the measurements in (**A**).

As an initial test for the utility of our GP saturation technique, we evaluated the impact of a focal lung injury on the collected functional metrics (Fig. 8). At baseline, the regional variations in pulmonary gas transport and efficiency at PEEP 0 cm H_2_O and 5 cm H_2_O exhibited the expected anterior-to-posterior gradient. Approximately 1 hour after HCl instillation, a ventilation defect became apparent in the posterior region of the injured right lung (white arrow in Fig. 8A). The reduced ventilation also manifested itself as a reduction in the contribution to the gas transport (Fig. 8B) and transport efficiency (Fig. 8C) in the posterior GP saturation volume. When a PEEP of 5 cm H_2_O was applied, the collapsed region of the lung was re-inflated. At the same time, the gas transport contributions and efficiency of the posterior-most lung region increased dramatically, even exceeding their respective baseline values, albeit at the expense of a decreased gas transport efficiency in the more anterior volumes.

## Discussion

This study set out to demonstrate the utility of regionally saturating the GP magnetization in selected airspaces of the lung as a means for assessing the contribution of that volume to the total pulmonary gas transport. In particular, we used the decrease in DP signal in the left heart following GP saturation relative to measurements without saturation as a non-invasive, conveniently accessible metric for quantifying regional lung function. In this initial implementation of our technique, we took advantage of the regional saturation feature in the scanner product software to position saturation slabs over the volume of interest and to destroy the GP magnetization with RF pulses centered at the xenon-129 gas resonance frequency prior to the start of data acquisition. Once imprinted, macroscopic GP saturation largely persists for the remainder of the breath hold; there are therefore no practical constraints on the available time for creating more complex patterns.

The degree to which the GP magnetization in the selected saturation volume is attenuated is a function of the applied effective flip angle and reaches it maximum for flip angles around 90°. Due to rapid xenon gas exchange between the alveolar airspaces and the lung parenchyma, saturation of the GP magnetization results in an almost instantaneous depolarization of xenon dissolved in the lung parenchyma as well. However, xenon magnetization farther downstream—such as in the major pulmonary veins, the left heart and the arteries—only washes out over several hundreds of milliseconds. Thus, to ensure that the steady state conditions associated with the created ventilation pattern had been established prior to the actual data acquisition, we first applied additional DP saturation pulses and a series of dummy RF excitations pulses with the same TR as during imaging. Residual GP and DP magnetization will be apparent in the saturation volume for saturation flip angles exceeding 90° (Fig. 2a), but will be 180° out of phase with the magnetization from lung regions unaffected by the GP saturation pulse. These two DP magnetization pools partially cancel each other out when they mix in the vasculature and the heart, further reducing the combined DP signal in these regions, although the total DP signal magnitude in the lung parenchyma is increasing (Fig. 2b). The RF saturation pulses in the product software could not be used to induce saturation flip angles in excess of 120° with our RF coil. Future refinement of our technique will permit the application of 180° inversion pulses within the saturation volume, which would double the sensitivity of the measurement relative to 90° saturation pulses, and which would be particularly useful for investigating smaller saturation volumes.

A coarse lung function profile can be calculated by incrementally moving a broad saturation slab position across the lung during consecutive measurements (Fig. 3). Due to the resulting crisp saturation slab profile, such an approach should be superior to the theoretically equivalent method of positioning narrow saturation bands over the lung, since the latter could introduce a slab thickness-dependent band profile into the analysis as an additional confounding factor.

In a supine rabbit, most of the gas uptake and transport occurs in the posterior regions of the lung. Thus, positioning the saturation slab so as to saturate the GP magnetization in the posterior volume of both lungs results in a large drop of the DP signal in the heart. This effect complicates the segmentation of the heart and impedes the quantification of small relative signal changes. Advancing the saturation slab in an anterior-to-posterior direction therefore usually yields superior results compared to advancing from posterior-to-anterior. Rotated saturation slabs might prove to be advantageous as a means of minimizing partial volume effects for one-sided lung pathologies. Although there is relatively little overlap between the lung parenchyma and the heart in axial projections in rabbits, the segmentation of the heart and vasculature can be greatly facilitated by saturating the GP magnetization in the anterior part of the lung, thus removing all DP background signal originating in the parenchyma (e.g. bottom rows of Fig. 3A). The segmented heart can then be used to isolate the same region within the images without regional saturation.

We showed that the change in xenon DP signal in the heart (ΔDP_H_) following regional GP saturation and the functional efficiency within the saturation volume are insensitive to operator-related variations in the manual segmentation process is low, and that measurement reproducibility in the same animal is high (Fig. 4A,B). In our measurements, the regional lung function in a healthy rabbit was not only impacted by its orientation (supine vs prone), but also by the inflation level of the lung—i.e., when during the respiratory cycle the breath hold was induced (EE vs EI), as well as the amount of PEEP applied. Flipping a rabbit from supine into a more natural, prone position resulted in an asymmetric redistribution of lung function, as demonstrated in Fig. 5. While in supine position, the contributions of the lung regions to gas uptake exhibited a strong, gravity-dependent gradient. In prone position, on the other hand, gas uptake appeared to be almost homogenously distributed in the vertical direction. However, this may be the result of larger tissue content in ventral vs. dorsal saturation slabs. We are currently investigating a tissue-volume-independent metric in which gas uptake and transport is normalized by local tissue volume measurements via proton MRI or CT or by using the relative contribution of the saturation slab to the total DP signal as a proxy. The functional efficiency gradient, on the other hand, followed gravity in both the supine and in the prone positions.

PEEP is an important tool for mitigating the risk of atelectasis and improving oxygenation in mechanically ventilated patients. Nevertheless, the effectiveness of PEEP and the optimal ventilator settings for maximizing its benefits while minimizing potentially detrimental side effects remain areas of great interest^35^. Fortunately, the impact of various lung inflation levels on pulmonary ventilation and gas transport are easily discernible with our technique (Figs 6 and 7). For one, the higher intrapulmonary pressure associated with increased lung inflation, either in the form of EE versus EI breath holds or as PEEP, compresses the heart and results in a smaller left ventricle size in the images. Although not directly quantifiable with our measurements, it stands to reason that at elevated alveolar pressure the pulmonary vasculature partially collapses and the blood flow rate is reduced^36^. The latter also results in a prolonged gas transit time from the alveolar airspaces to the heart; for the selected flip angle and TR, our technique is very sensitive to any such time-delays. As a consequence, the gas transport efficiency varies dramatically throughout the respiratory cycle: up to a factor of ~7 (Fig. 7B) between PEEP 0 and PEEP 10 cm H_2_O for breath holds at EE. Figure 7A also indicates pressure-dependent changes in the spatial distribution of the gas transport contributions. However, this interpretation could be misleading, as we advanced the positions of the saturation slabs in fixed increments and not with respect to their anatomical location. For future studies, it might be advantageous to distribute a fixed number of GP saturation slabs evenly across the entire lung volume.

While it can be expected that the application of PEEP always leads to large reductions in gas transport efficiency in healthy lungs, the situation could be altogether different in injured or diseased lungs. Under such circumstances, higher intrapulmonary pressure can drastically increase the lung volume involved in gas exchange, more than compensating for decreased regional functional efficiency. As an initial demonstration of our method’s sensitivity to changes in gas transport in an acutely injured lung imaged with and without PEEP, we used a rabbit acid aspiration model. We found that lung function is spatially redistributed following administration of the acid (Fig. 8), most likely due to perfusion changes in response to the insult. Of particular interest, however, was the observation that the injury-induced ventilation defect in the right posterior lung resulted in greatly reduced gas transport efficiency in the posterior-most slice of the lung. A PEEP of 5 cm H_2_O re-inflated the collapsed lung and caused the gas transport efficiency of that region to spike, even exceeding its baseline value, but reduced functional efficiency in the presumably less severely injured anterior lung volumes. This finding indicates that there should exist an identifiable, inflation-dependent maximum in gas-transport efficiency, and emphasizes the large potential benefit of using our method to find optimal PEEP settings in acute lung injury. However, it is important to note that the current efficiency measurements were based on 2D acquisitions, and so did not take GP concentration changes due to lung expansion in the third dimension into account. In the future, we will include 3D volumetric scans to our study protocol to correct for differences in ventilated lung volume at different PEEP levels. Knowledge of this parameter will also allow us to investigate whether a change in functional lung efficiency is partially compensated for by an offsetting change in lung volume.

In this study, we showed that HXe DP MRI in conjunction with regional GP saturation can be used to gain additional insights into lung function in the form of pulmonary gas transport and its efficiency. The difference between these parameters and existing hyperpolarized-gas techniques is that regional deficiencies in ventilation, gas exchange, or perfusion are all captured simultaneously and integrated into one metric. In contrast with existing DP xenon MRI techniques, reported values do not reflect only the xenon distribution in the parenchyma, but instead the actual xenon transport. While existing methods can localize regional abnormalities, they cannot quantify the impact of any given abnormality on overall lung function or provide much insight into potential compensatory mechanisms by comparatively healthy lung regions. Although it can be expected that the measured xenon gas exchange dynamics will differ from those for oxygen and carbon dioxide, they are susceptible to the same pathological changes in physiology, e.g. abnormal ventilation patterns, surface-to-volume ratio, septal wall thickness, etc. Therefore, regionally abnormal lung function affecting oxygen and carbon dioxide exchange should also be reflected in the xenon gas exchange and transport in a similar manner. In disease, some parts of the lungs may be responsible for most gas exchange function, while others may contribute only minimally or be functionally silent. Information about this condition could improve the management of patients with chronic lung diseases (e.g. in the evaluation for lung transplant or lung reduction surgery), and aid preparations for lung cancer resection. In patients with acutely injured lungs, smaller portions of parenchyma (the “baby lung”) perform all gas exchange^37^. In this situation, mapping gas uptake and transport with our GP saturation technique could provide a better marker of disease severity than arterial blood gases, which are notoriously inaccurate and heavily affected by regional variability in lung performance. Although our method in its current form already seems to be very susceptible to alterations in lung function due to positioning and acute lung injury, more extensive studies in both animals and human subjects will be required to optimize its sensitivity and evaluate its full potential.

## Data Availability

The datasets generated and/or analysed during the current study are available from the corresponding author on reasonable request.
